# A Novel Vibrating Capsule Treatment for Constipation: A Review of the Literature

**DOI:** 10.7759/cureus.52943

**Published:** 2024-01-25

**Authors:** Tesingin D Uwawah, Basil N Nduma, Stephen Nkeonye, Davinder Kaur, Chukwuyem Ekhator

**Affiliations:** 1 Internal Medicine, Cherubin Family Health Care, Brooklyn, USA; 2 Internal Medicine, Medical City Hospital, Denton, USA; 3 Research and Development, University of Texas MD Anderson Cancer Center, Houston, USA; 4 Internal Medicine, Medical City Hospital, North Hill, USA; 5 Neuro-Oncology, New York Institute of Technology, College of Osteopathic Medicine, Old Westbury, USA

**Keywords:** constipation, chronic constipation, irritable bowel syndrome, functional constipation, dyssynergic defecation, rectal evacuation disorders, outlet obstruction, slow colon transit, vibrating capsule

## Abstract

Constipation is a pretty common and sometimes complicated health condition around the world which is characterized by an inability to have regular bowel movements. In response to this worrying trend, various pharmacological and non-pharmacological interventions have been embraced to seek to produce promising outcomes, yet patient dissatisfaction continues to be reported. The main aim of this review paper was to determine the effectiveness and safety of the vibrating capsule in treating constipated patients. The key databases that were consulted to get articles on this subject include Google Scholar, Embase, and PubMed. Specific keywords were used in the database search to get the relevant articles. Based on the exclusion criterion, articles that were excluded include conference abstracts, commentaries, preclinical research articles, articles where full texts were inaccessible, and those that had been published in a language other than English. From the results, the safety profile of the vibrating capsule suggests that the intervention is generally well-tolerated, with only mild and transient side effects or adverse events noted, including abdominal discomfort and sensations of mild vibration. However, the impact of these adverse events (although mild to moderate) on the efficacy of the capsule remains unknown, an area requiring further scholarly attention in the future. Concerning the efficacy of the intervention, most studies were found to affirm that the vibrating capsule enhances the physiologic effects of meals and waking on bowel movements, but the need for providers in clinical environments to note the interplay between the number of vibrations and the effectiveness of the capsule or onset of complete spontaneous bowel movements could not be overemphasized. In conclusion, this paper established that the vibrating capsule is an effective and promising technology through which constipated patients could be treated while experiencing minimal or no adverse events, but future research efforts ought to seek to uncover the interplay between the mechanism of action of the capsule and any moderating role played by factors internal or external to patients, including their emotional, mental, and psychological statuses, as well as the type and quantity of food consumed before and after the vibration sessions.

## Introduction and background

In clinical practice, major gastrointestinal disorders have been diagnosed, and constipation is one of them, especially in Western countries. In particular, close to 20% of the population suffers from this condition, implying that the disorder reflects a substantial utilization of healthcare services [1]. Although constipation forms one of the most debilitating health conditions, its pathophysiology remains multifactorial and complex. Some of the aspects that add to this complexity include psychological distress, lifestyle habits, genetic predisposition, and disturbance in colonic transit [1]. The first line of therapy has been avowed to include patient education on the criticality of lifestyle adjustments or changes such as sports activity state and diet, reflecting a non-pharmacological approach. In situations where the non-pharmacological approach is ineffective, pharmacological options have been explored and utilized, including the use of serotonergic agonists, secretagogues, and laxatives [2]. In further scenarios where the pharmacological options are ineffective, the surgical approach is used as the definitive solution for constipation. Thus, it can be inferred that a serious issue accruing from constipation includes a decreased quality of life among patients, as well as substantial medical care costs [3].

Globally, constipation is estimated to stand at about 12% to 19% in prevalence [2]. Compared to Asian countries, the condition is more prevalent in European countries and North America. Indeed, these trends or patterns are linked to the probable role of the environment and dietary habits [4]. Notably, the basic definition of constipation is that it involves a reduction in the number of defecations each week. Also, the condition involves multiple other symptoms, including the necessity for digital disimpaction, hard stools, failed or elongated attempts to defecate, straining, abdominal bloating, and incomplete evacuation [5]. Given its etiology, constipation may occur in two major forms, which include primary constipation and secondary constipation. On the one hand, primary constipation includes functional constipation, constipation-predominant irritable bowel syndrome (IBS-C), and slow transit constipation including functional defecation disorders, neuropathy, and myopathy [6]. On the other hand, secondary constipation may be due to neurological disorders, primary colonic disorders (such as proctitis and cancer), medications (such as opiates or calcium channel blockers), and metabolic disorders (such as hypercalcemia) [6].

Among patients, constipation has been asserted to yield a notable decrease in their health-related quality of life because of the psychological distress and physical symptoms with which it comes. Mostly, patients with constipation have been asserted to complain about urine retention, sexual dysfunction, and dyspareunia [7]. Also, in the case of chronic constipation, social activities and work productivity might be limited. The situation is exacerbated by the position that constipation diagnosis and treatment pose a significant economic burden, with testing for the condition alone costing approximately $7 billion each year [8]. Thus, the associated decrease in the quality of life, costs of medical care, and the high prevalence of constipation make it a serious issue.

A notable point is that most of the recent surveys hold that more and more constipated patients continue to express dissatisfaction with current treatments. Particularly, as many as 47% of constipated patients have been asserted not to be completely satisfied with their current treatments for constipation, hence a growing pattern in the interest in new therapies [9]. Also, direct intestinal mechanical stimulation or stool dispersion in a hollow lumen has been documented to reflect a promising avenue for constipation therapy due to their associated ability to facilitate movement. Previously, studies have used different types of external vibration devices, including an external vibrating belt or a vibrating platform, with the results holding that constipation could be mitigated using vibration devices [10]. Indeed, these investigations have centered on a non-invasive oscillation platform that induces low-intensity whole-body vibration in a quest to reduce the severity of the symptoms in individuals diagnosed with chronic functional constipation, with the findings in these patients avowed to depict more promising patient outcomes than in the control group that is not exposed to the low-intensity whole-body vibration approach. In a quest to build on such electro-mechanical devices, deviating from external vibration, the vibrating capsule has emerged, charged with the stimulation of the walls of the intestines through local contact, aimed at stool movement stimulation, yet some studies indicate that the need for further testing of this hypothesis could not be overstated [11]. 

Evolving as a miniaturized and novel capsule device, the vibrating capsule is an alternative non-pharmacological treatment approach for dysmotility in the gut. Indeed, an electromagnetic signal activates the capsule, which has an activation code arising from the base unit. The code entails both the duration and timing of each capsule’s vibration [12]. Importantly, most studies do not report severe adverse events, but concerns such as proctalgia (pain arising from pelvic floor muscle spasms), flatulence (passing gas), blood in stool, vomiting, abdominal discomfort, abdominal distension (swollen abdomen), abdominal pain, diarrhea, and nausea have been listed [13]. Therefore, whether adversities (if any) that might be associated with the vibrating capsule tend to outweigh the perceived beneficial effects of this novel, miniaturized approach for treating constipation or otherwise is an area worth clarifying through additional scholarly investigation or research. Conducted from a literature review perspective, this study aimed to find out the efficacy and safety of the vibrating capsule as a treatment modality in constipated patients, gaining insights from recent scholarly studies centering on this subject.

## Review

Materials and methods

**Figure 1:**
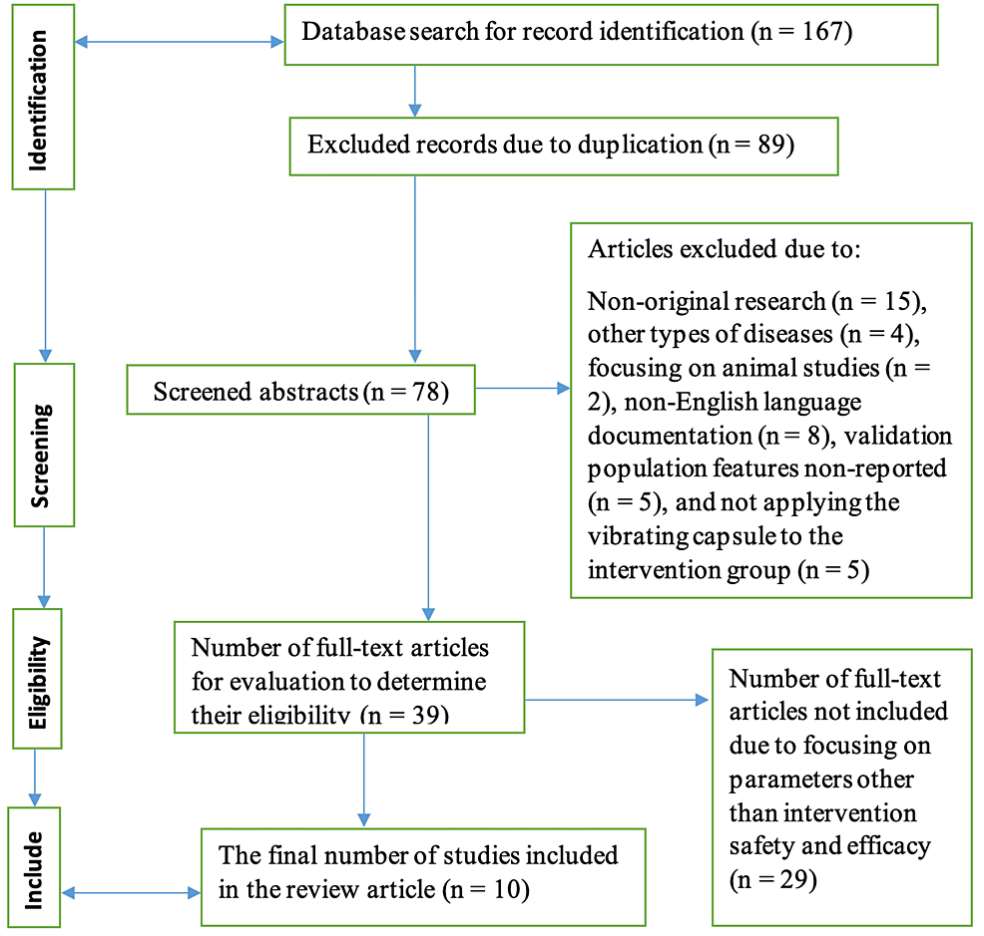
Preferred Reporting Items for Systematic Reviews and Meta-Analyses (PRISMA) flow diagram showing an illustration of the article selection criteria

To search the materials for this review paper, some of the utilized databases included Google Scholar, Embase, and PubMed. In the research query, certain combinations of keywords were embraced. They included slow colon transit, outlet obstruction, rectal evacuation disorders, dyssynergia defecation, functional constipation, irritable bowel syndrome, chronic constipation, and vibrating

capsules. The findings were limited to relevant articles published in the English language. Also, there were no restrictions regarding the publication dates of the articles that the selected studies might have cited in their respective subsections. However, the primary studies should have been conducted between 2010 and 2023 to contribute to the recency and relevance of the current state-of-the-art. To also ensure that more eligible articles were centered on, there was a review of the references in all studies that were included. Thus, each research team member would review each article independently to ascertain its inclusion or otherwise, as informed by the exclusion and inclusion criteria developed previously. In scenarios involving some degree of disagreement in the research team, they would be resolved via discussions until a consensus was reached. Notably, conference abstracts were not included in the review paper. Other articles excluded from this investigation or review paper included commentaries, preclinical research articles, articles where full texts were inaccessible, and those developed in a non-English language. In articles that were included in the review, some of the details that were extracted included the study methodology, details of interventions and controls, and study population and demographics. The following Figure 1 is a Preferred Reporting Items for Systematic Reviews and Meta-Analyses (PRISMA) flow diagram that offers a research summary of the article selection criteria [14-23].

Results

Table 1 below shows results of sample studies and clinical implications. 

**Table 1:** Results of sample studies and clinical implications

References	Author(s)	Year	Study Aim	No. of Participants	Results	Clinical Implications
[14]	Zhu et al.	2022	To assess the safety and efficacy of smartphone-controlled vibrating capsules in individuals diagnosed with functional constipation.	107	The vibrating capsule promotes defecation while ameliorating disease symptoms and improving patient quality of life, coming also with sustained efficacy. Abdominal discomfort emerged as the most common adverse event with which the vibrating capsule would be associated.	The study ascertained the efficacy and safety of the vibrating capsule, but whether the findings could hold uniformly in varying clinical settings remained unclear.
[15]	Rao et al.	2020	To evaluate the oral vibrating capsule’s mechanistic effects in patients diagnosed with chronic idiopathic constipation in terms of the circadian rhythm, complete spontaneous bowel movements, and the onset of vibrations.	250	The vibrating capsule was found to increase complete spontaneous bowel movements by enhancing the physiologic effects of waking and meals, as well as circadian rhythm augmentation.	An increase in complete spontaneous bowel movements was avowed to arise from increased vibration sessions.
[16]	Saeed et al.	2023	To find out the eligibility of an orally ingested vibrating capsule in the management of chronic constipation	601	In the three randomized controlled trials involving the vibrating capsule group and placebo, the change in the complete spontaneous bowel movement from baseline in both groups was not statistically significant, as well as the incidence of adverse events. However, there was increased vibration sensation in the vibrating capsule group.	The study suggested that evidence about the effectiveness of the vibrating capsule remains unclear.
[17]	Rao et al.	2023	To establish the safety and efficacy of the vibrating capsule in individuals diagnosed with chronic constipation.	904	From baseline, with eight weeks of investigation, the study pointed out a significant improvement in the vibrating capsule group relative to secondary endpoints that involved measures of the quality of life, stool consistency, and straining compared with placebo. On safety, mild adverse events were reported, mostly gastrointestinal-related.	Compared to the placebo, this study depicted the superiority of the vibrating capsule concerning its associated promising role of a better quality of life and bowel symptom improvement.
[18]	Rao et al.	2022	312	To examine the safety and efficacy of the vibrating capsule in individuals diagnosed with severe chronic idiopathic constipation (CIC)	In the findings, the vibrating capsule did not yield severe adverse events or diarrhea, with the most common adverse events being abdominal discomfort in 2.25% of the group and a sensation of mild vibration in 11.2% of the group. On effectiveness, the vibrating capsule group experienced significant improvements in the quality of life than placebo, as well as significantly greater improvements in the complete spontaneous bowel movements, quality of life, consistency, and straining effort in the vibrating capsule group compared to the placebo.	The study revealed that the treatment approach using the vibrating capsule is generally safe.
[19]	Rao et al.	2018	245	To determine the effects of the vibrating capsule on complete spontaneous bowel movements among patients diagnosed with CIC through the use of two paradigms of vibrating capsule activation.	There was a significant correlation between the frequency of occurrence of the complete spontaneous bowel movements and the time of pre-defined vibration in the single vibration group.	The timing of vibration of the vibrating capsule could be seen to determine the number and percentage of complete spontaneous bowel movements.
[20]	Quigley et al.	2017	188	To determine the effectiveness of the vibrating capsule in constipation management.	In patients diagnosed with CIC of moderate to severe intensity, a vibrating intraluminal capsule comes with dose-dependent relief with no serious adverse side effects reported.	With only one subject in either study discontinuing due to an adverse event, the study ascertained the promising role of the vibrating capsule in improving symptoms of constipation but, in clinical environments, it is crucial to determine the specific dose to apply and also the time of the pre-defined vibration to ensure optimal patient outcomes are factors worth considering.
[21]	Feinstein et al.	2021	40	To investigate the effect of an ingested vibrating capsule on neural responses to gastrointestinal sensation through perceptual response, stomach, and brain quantification.	The investigation suggested that a minimally invasive form of mechanosensory gastrointestinal stimulation alters gut feeling perception reliably. Also, vibrating stimulation in the stomach would be observed to evoke brain responses in its midline parieto-occipital electrodes at both late and early periods after capsule vibration.	The study ascertained that there is an intersection between the vibrating capsule and neural responses in the brain system, thus regulating gut feelings and contributing to constipation management.
[22]	Sharma et al.	2021	171 references	To assess the perspective of patients on the effect of the vibrating capsule as a treatment option for constipation.	The results demonstrated that novel smartphone-based applications such as the vibrating capsule could aid in tracking symptoms of constipation, with the efficacy of this treatment modality documented to be complemented by the utilization of other interventions such as lifestyle and diet changes.	Whereas the vibrating capsule’s efficacy was ascertained by the authors, a multidisciplinary approach is key to realizing optimal patient outcomes.
[23]	Medical Xpress	2014	26	To determine the effectiveness of the vibrating capsule as a non-pharmacological intervention for constipation treatment.	In the results, the vibration capsule was found to almost double bowel movements each week for individuals diagnosed with CIC and also constipation-predominant irritable bowel syndrome.	The superiority of the vibrating capsule could be seen in terms of its promising ability to decrease constipation symptoms such as incomplete evacuation and difficulty in passing stools, as well as increased spontaneous bowel movements, with its safety profile ascertained via minimal side effects. In clinical contexts, thus, the vibrating capsule could be utilized for candidates of the same.

In one of the recent studies [14], the efficacy and safety of the vibrating capsule in patients diagnosed with functional constipation were examined. One hundred seven participants aged between 18 and 74 were assigned randomly to the placebo treatment (n = 54) or the vibrating capsule (n = 53). In this study, a significantly high responder rate was achieved. The results demonstrated that the vibrating capsule promotes defecation, steers improvements in the quality of life, ameliorates symptoms of constipation and comes with minimal adverse events, with the most common adverse event reported being abdominal discomfort. As such, the safety and efficacy of the intervention was ascertained. However, whether these results would still hold even in situations where the participants differed significantly in terms of demographic features remains unclear. Similarly, whether the subject of disease severity could alter the efficacy of the vibrating capsule remains unknown.

In another study, the oral vibrating capsule was examined in individuals diagnosed with chronic idiopathic constipation (CIC). Temporal relationships among aspects of the circadian rhythm and complete spontaneous bowel movement [15], motivated the need to inform the effectiveness of this technique. With a focus on 250 participants, the results demonstrated that the vibrating capsule augments the circadian rhythm and also enhances the physiologic effects of waking and meals, leading to increased complete spontaneous bowel movements. Thus, the effectiveness of the vibrating capsule is ascertained in this study. However, whether the degree of safety and the amount of time between the onset of constipation in patients and the introduction of the vibrating capsule could impact the treatment effectiveness remains unknown. Also, whether adverse events, if any, could differ in magnitude based on the aforementioned factors is yet to be clarified.

In yet another study to examine the impact of the vibrating capsule on the management of chronic constipation [16], an electronic database with data examined from February 27, 2013 up to 2023 was used. A meta-analysis was done using RevMan. The results demonstrated no statistically significant difference between the vibrating capsule group and placebo, even though an increase in the vibration sensation arose in the vibrating capsule group. On the safety factor, however, the superiority of the vibrating capsule was documented. Specifically, the vibrating capsule was avowed to be well-tolerated and safe whereby there were no significant harmful effects noted. In the future, however, the study pointed to the need for additional large-scale randomized controlled trials to ascertain the vibrating capsule’s safety and efficacy in persons diagnosed with functional constipation. Another direction to highlight for further scholastic clarification is whether factors such as the duration of treatment and the frequency of treatment could alter the safety and effectiveness of the vibrating capsule.

Still, in a quest to contribute to the current state-of-the-art in academia concerning efforts to mitigate the unmet need of patients seeking effective chronic constipation treatment, the safety and efficacy of the vibrating capsule were investigated [17]. A double-blind, placebo-controlled trial in which the randomization of patients saw them receive either a vibrating or placebo capsule once each day for five days within the respective weeks, an intervention stretching for eight weeks. In this study, the superiority of the vibrating capsule over the placebo was documented, including significantly greater improvements in secondary endpoints of measures of quality of life, stool consistency, and straining. Also, with only mild adverse events related to the gastrointestinal system observed, the vibrating capsule was found to be relatively safe. However, whether any pre-existing conditions could have impacted the effectiveness of the vibrating capsule and also its safety remained unaddressed in the study. 

Furthermore, in the context of patients with CIC, the effectiveness and safety of the vibrating capsule compared to the placebo were investigated [18]. In this study, 312 CIC patients were enrolled, including 86 participants in the placebo group and 89 participants in the vibrating capsule intervention group. A significantly higher complete spontaneous bowel movements were observed in the vibrating capsule group compared to the placebo. Also, the superiority of the vibrating capsule over the placebo was reported regarding parameters such as quality of life, stool consistency, and straining effort. It is further notable that the vibrating capsule intervention was affirmed to be generally safer, with no diarrhea or severe adverse events noted. In this group, the most common adverse events were abdominal discomfort and a sensation of mild vibration. The vibrating capsule was well tolerated, and yielded significant improvements in constipation. However, it is yet to be investigated whether or not the severity of CIC was likely to impact the level of effectiveness of the vibrating capsule, as well as its safety, hence the need for more scholarly attention around this area to document the efficacy and safety of the intervention with certainty [18]. 

The impact of the vibrating capsule on complete spontaneous bowel movements was also investigated from the perspective of two paradigms of vibrating capsule activation [19], with a particular focus on individuals diagnosed with CIC. The inclusion criterion for this study included patients who fulfilled Rome III criteria for constipation. Patients who met the inclusion criteria were added to two double-blind, sham-controlled investigations. The study was organized in such a way that a single vibration session was implemented for 182 patients in study 1 and multiple vibration sessions per day were implemented for 63 patients in study 2. The study concluded that the vibrating capsule increases the number of complete spontaneous bowel movements compared to the sham. It was therefore inferred that the mechanical vibration of the colon reflects a key mechanism of action of the vibrating capsule relative to its eventual induction of bowel movements, a pattern that would also see peristalsis and colonic biorhythm augmented. This study thus revealed the vibrating capsule as a unique, non-pharmaceutical modality that may yield promising results in terms of efficacy, with the factor of the time of pre-defined vibration playing a moderating role in shaping this state of effectiveness [19].

Two dosing regimens of the vibrating capsule were also compared via an aggregation of data from two studies, with inclusion criteria being subjects with CIC who also satisfied Rome III criteria for constipation, and both groups exhibiting comparable exclusion and inclusion criteria [20]. In the first study 163 subjects ingested two capsules each week for eight weeks at the site, meanwhile, in the second study, 25 subjects ingested five capsules each week for eight weeks at home. At the end of the study, it was realized that both studies recorded an increase in the number of complete spontaneous bowel movements. Also, there were significant improvements in different symptoms of constipation, including bloating, straining, and consistency of stool. From the safety analyses, the investigation affirmed an excellent safety profile of the vibrating capsule, with no serious adverse events linked to the device reported in either investigation, with only 13 moderate gastrointestinal adverse events reported in the first study. As such, the study led to an inference that in the wake of moderate to severe CIC, a vibrating intraluminal capsule offers dose-dependent relief, with no serious adverse events incurred. However, a point worth highlighting is that in the selected study, part of the enrolled subjects ingested the capsule at the site while others ingested it from home. Hence, whether these variations in the environmental conditions under which the interventions took place might have posed an impact on the efficacy of the capsule remained unknown. Also, whether adherence to the intervention for subjects who took the capsule at home was ascertained remained unclear. 

To understand the correlation between the vibrating capsule’s mechanism of action and brain responses, as well as the secondary impact on gut responses, an investigation consisting of 40 participants was conducted, with insights gained from electrogastrogram (EGG) and EEG signals [21]. The investigation ascertained the efficacy of the vibrating capsule in altering brain activity and neural responses, which would then impact gut feelings, hence a promising non-invasive approach to constipation management. However, it is worth noting that different patients could exhibit variations in their psychological and thought processes at any given moment, and so whether these variations could impact how the vibrating capsule stimulates the brain and other neural processes that would, in turn, regulate bowel movements (thus constipation management) is an area that remained unaddressed in the study, hence the criticality of future studies focusing on such a research gap.

From a systematic review perspective, the effectiveness of the vibrating capsule in a multidisciplinary treatment context was evaluated [22]. In this study, the central purpose was to uncover the safety profile and effectiveness of the vibrating capsule relative to the management of constipation. One hundred seventy-one references were selected and included in the review, having confirmed their relevance to the study’s scope. The study affirmed that an ingested oral vibrating capsule ensures alterations in the circadian rhythm and also steers improvements in constipation via the induction of more complete spontaneous bowel movements. Therefore, the results can be seen to contribute significantly to the current state-of-the-art, proceeding further to increase an understanding of the importance of combination therapy in yielding optimal patient outcomes, including accompanying the vibrating capsule intervention with psychological interventions (such as psychological therapy, hypnotherapy, and cognitive behavioral therapy) and habit training (such as bowel or pelvic floor training). However, two key areas remain unaddressed. The first key factor is how to determine the type of complementary therapy to embrace alongside the vibrating capsule. The second key factor is how to determine candidates for the combinational therapy versus those for stand-alone vibrating capsule intervention, hence the need for more scholarly attention.

In another study involving 26 patients, the safety profile and effectiveness of the vibrating capsule were investigated [23], with the aim of informing the decision for or against recommending (or prescribing) this capsule. In this study, the subjects used the vibrating capsule two times each week for two weeks with no other intervention. This was proceeded by use of the vibrating capsule along with laxative. A daily bowel movement questionnaire was administered through the study. In the results, it was evident that the vibrating capsule is effective in such a way that it almost doubles bowel movements weekly in persons diagnosed with constipation-predominant irritable bowel syndrome and CIC. Also, the safety profile of the capsule was documented, with minimal side effects reported. As such, it can be inferred that this capsule yields better quality of life among candidates, proving to be a promising non-pharmacological intervention. However, it is worth contending that the causes of constipation differ variably. Therefore, an intersection between the type of risk factor and the effectiveness and safety of the vibrating capsule remained unclear in the above-mentioned study, pointing to the need to delve further into this research area.

Discussion

Designed to stir the colon to action, the vibrating capsule can be seen to nearly double the ability of constipated patients to defecate more normally without necessarily prompting pharmaceutical interventions. However, when it comes to the efficacy and safety of this intervention, mixed outcomes are evident. In most studies, the safety profile of this intervention was documented. However, the technique has not gone without attracting some adverse events or side effects, even though these events were mostly documented as mild and transient. In the wake of these mixed outcomes, an additional point to note is that the role of certain environmental and patient-specific factors in shaping the effectiveness of the strategy and its safety profile remains unknown in the majority of cases.

In situations where participants were exposed to the vibrating capsule for a period spanning eight weeks, the number of complete spontaneous bowel movements was affirmed to almost double when compared to the groups taking a placebo. On adverse effects, the intervention is generally well-tolerated, with no serious adverse events reported in the majority of the cases. Even in scenarios where serious adverse events were reported during the vibrating capsule intervention or implementation, most incidences were unrelated to study treatments, including encounters such as pelvis fractures and anxiety attacks. Adverse events related to the study treatments (such as diarrhea and nausea) were generally low to moderate severity. It can thus be concluded that adverse effects or events accruing from the vibrating capsule are low, with most of these events being mild and transient. Throughout this systemic review, the literature fails to document whether the effectiveness of the capsule would operate almost uniformly for patients who experience some adverse events versus those who experience no adverse effects or whether the mechanism of action and effectiveness of the intervention would to be impaired to some degree when these events occur, especially concerning the impact of the intervention on the quality of life of patients. In the future, such an area is worth examining comprehensively to ensure that if adverse events impair the effectiveness of the vibrating capsule to some degree, relevant adjustments to the goals or plans of action in clinical environments could be made to ensure consistent wellness on the patient’s side even in the wake of any mild and transient side effects of the capsule.

This review paper has also noted the impact of the vibrating capsule on the circadian rhythm and complete spontaneous bowel movement. Indeed, most studies contend that the capsule comes with a higher proportion of complete spontaneous bowel movements, either up to about three hours beyond the vibration time or during the vibration itself. Within such a time window, the reviewed articles majorly contend that there tends to be an enhanced and statistically significant effect of the active capsule. What is also worth highlighting is that the timing of the vibration sessions exhibits a distinct trend correlating with the onset of complete spontaneous bowel movements. This correlation particularly suggests that the peaks of the complete spontaneous bowel movements tend to be realized mostly close to the vibrating capsule’s vibration sessions, including close to mealtimes around noon, upon awakening, and in the mornings. The implication for providers in clinical environments is that in situations where the vibrating capsule is adopted, it is critical to understand this relationship between the vibration session and the onset of complete spontaneous bowel movements in order to adequately time the intervention and prevent interaction with other treatments. Despite these highlighted informative observations, this review paper established certain gaps requiring further investigation. For instance, most of these scholarly positions do not account for the type and quantity of food before or after the vibration sessions. Whether this factor could alter the effectiveness of the vibrating capsule remains unknown. Also, the scholarly studies fail to document specific clinical environments in which external conditions might enhance or impair the activity of the vibrating capsule, including factors such as the temperature, time of the day, and whether crowding (or otherwise) might affect the onset of complete spontaneous bowel movements following vibration sessions. Indeed, these are noteworthy areas in need of further research attention in the future. Similarly, different patients present to clinics with different disease symptoms, varying disease severity and different psychological state of mind or perception of their disease. Thus, the intersection between mental, emotional, and psychological states internal to the patient and the onset of complete spontaneous bowel movements is yet to receive an in-depth analysis, thus the need for future scholarly investigations centered on these areas.

## Conclusions

In summary, this review paper aimed to uncover the efficacy and safety of the vibrating capsule as an intervention for constipated patients, including individuals diagnosed with functional constipation and those experiencing chronic idiopathic constipation. From the scholarly sources that were consulted, mixed outcomes arose. From the perspective of the safety profile, most studies contended that the vibrating capsule is generally well-tolerated with no common cases of severe adverse events. In scenarios where adverse events are reported, including abdominal discomfort and sensations of mild vibration, the majority tend to be of low or mild and transient magnitude. However, whether these mild adverse effects have an impact on the effectiveness of the capsule remained unknown. Concerning the effectiveness of the intervention, the review outcomes suggested that the vibrating capsule is associated with enhancements in the normal physiologic effects of meals and waking on bowel movements. The number of vibrations was also found to impact the effectiveness of the capsule, with at least two vibration sessions per day proving superior leading to significant relative increase in the number of complete spontaneous bowel movements. However, as mentioned in the discussion, there is a need to examine the interplay between factors internal and external to the patient in the clinical environment to help understand clearer trends concerning the efficacy of the intervention. Overall, however, the observation that the vibrating capsule improves the physiologic effects of waking and meals on bowel movements and also comes with a favorable safety profile leads to an affirmation that the highly promising technology may be beneficial to constipated individuals who are less responsive to pharmaceutical interventions - but the need for placebo-controlled, sham, larger, or better-designed studies to confirm these inferences should not be overstated. 
